# Optical coherence microscopy in 1700 nm spectral band for high-resolution label-free deep-tissue imaging

**DOI:** 10.1038/srep31715

**Published:** 2016-08-22

**Authors:** Masahito Yamanaka, Tatsuhiro Teranishi, Hiroyuki Kawagoe, Norihiko Nishizawa

**Affiliations:** 1 Department of Quantum Engineering, Nagoya University, Furo-cho, Chikusa-ku, Nagoya, Aichi, 464-8603, Japan

## Abstract

Optical coherence microscopy (OCM) is a label-free, high-resolution, three-dimensional (3D) imaging technique based on optical coherence tomography (OCT) and confocal microscopy. Here, we report that the 1700-nm spectral band has the great potential to improve the imaging depth in high-resolution OCM imaging of animal tissues. Recent studies to improve the imaging depth in OCT revealed that the 1700-nm spectral band is a promising choice for imaging turbid scattering tissues due to the low attenuation of light in the wavelength region. In this study, we developed high-resolution OCM by using a high-power supercontinuum source in the 1700-nm spectral band, and compared the attenuation of signal-to-noise ratio between the 1700-nm and 1300-nm OCM imaging of a mouse brain under the condition of the same sensitivity. The comparison clearly showed that the 1700-nm OCM provides larger imaging depth than the 1300-nm OCM. In this 1700-nm OCM, the lateral resolution of 1.3 μm and the axial resolution of 2.8 μm, when a refractive index was assumed to be 1.38, was achieved.

Recent studies in life science have revealed that various biological features in organisms, such as tissue-specific morphology and physiology, are impaired in conventional cell culture on flat and hard substrates^1^,^2^. To understand natural biological behaviors and architectures in organisms, it is significantly important to observe the inside of organisms or its tissue specimens with high spatial resolution in three dimensions (3D). Current rapid advancements of cell culture technologies have made it possible to provide a variety of ways to create biological environments to produce artificial tissue specimens, which can closely mimic biological features in organisms^3^,^4^,^5^. These facts leave no room for doubt that it will become more and more important in biomedical studies to improve the imaging depth of 3D high-resolution imaging. Currently, multiphoton excited fluorescence microscopy has been widely used for high-resolution deep-tissue observations^6^,^7^,^8^. Although the fluorescence technique is a powerful tool to observe specific sites in deep regions of thick biological specimens with cellular-level spatial resolution, it requires labeling of specimens with endogenous dyes or genetically encoded fluorescent proteins, which usually has large molecular weight and may alter natural biological activities.

To visualize natural conditions of specimens, it is desirable to use imaging techniques which do not need any physical or chemical treatments. Optical coherence tomography (OCT) is well recognized as a non-invasive label-free imaging technique with an axial resolution of 1–15 μm^9^,^10^,^11^. Since the first report of OCT in 1991^12^, OCT has been exploited for diverse applications in biomedical studies due to the excellent imaging capability^13^,^14^,^15^,^16^. Optical coherence microscopy (OCM) is an imaging modality based on OCT and confocal microscopy, which realizes high spatial resolution in 3D^17^. The combination of OCT and confocal detection schemes also helps to improve the image contrast by the enhanced rejection capability of signals from out-of-focus. So far, OCM techniques have been successfully applied to visualize small details in various biological samples, such as myelin fibers and cerebral cortex in brain specimens^18^,^19^,^20^,^21^. One of the current critical issues in OCM is its shallow imaging depth, which is limited by light attenuation mainly due to multiple light scattering in samples and light absorption of water as well as in the case of OCT^22^. Because OCM provides the imaging capability with high spatial resolution in 3D, the improvement of the imaging depth in OCM would be greatly beneficial to a wide variety of biological investigations, such as brain studies.

Although the 800-nm spectral band, which is one of the main wavelengths used for OCT and OCM, offers excellent spatial resolution^11^,^23^,^24^, it is difficult to access deep regions of turbid scattering tissues due to the strong attenuation of light by the high scattering coefficient in samples. One of the major efforts to overcome the limitation of the imaging depth is to use longer wavelength regions, since the scattering coefficient monotonically decreases when the wavelength becomes longer. OCT and OCM techniques operating at the 1000–1300 nm spectral region have been developed to achieve large imaging depth^25^,^26^,^27^,^28^,^29^,^30^. Recently, 1300-nm swept-source (SS) OCT realized 2.3 mm imaging depth in the observation of a mouse brain by using a 1300-nm vertical cavity surface emitting laser^31^. In addition, it was also reported that 1300-nm OCM enabled 3D high-resolution imaging of a mouse brain at a depth up to ~1.3 mm^20^. Recent investigations on improving the imaging depth revealed that the 1700-nm spectral band offers promising prospects for deep-tissue imaging of turbid scattering tissues^32^,^33^. This is owing to the less attenuation of light in the 1700-nm spectral band than that of the 800–1300 nm spectral band, which arises from the existence of the local minimum of the absorption coefficient by water and the reduced scattering coefficient in the wavelength region. Although the lower scattering coefficient in the 1700-nm spectral band results in the decrease of the backscattering efficiency, currently-reported 1700-nm OCT successfully demonstrated that the use of the 1700-nm spectral band enables to enhance OCT imaging depth in the observation of not only industrial materials and teeth samples, which are highly scattering samples with low water content, but also animal tissues such as brain samples^34^,^35^,^36^,^37^,^38^. So far, we have developed a high-power supercontinuum (SC) source in the 1700-nm spectral band for OCT and have demonstrated high-resolution OCT imaging with enhanced imaging depth^34^,^35^,^36^. In this work, we developed OCM by using the high power SC source in the 1700-nm spectral band and performed the 1700-nm and 1300-nm OCM imaging of animal tissues to demonstrate that the 1700-nm OCM has the benefit in terms of the imaging depth.

## Results

**Figure 1:**
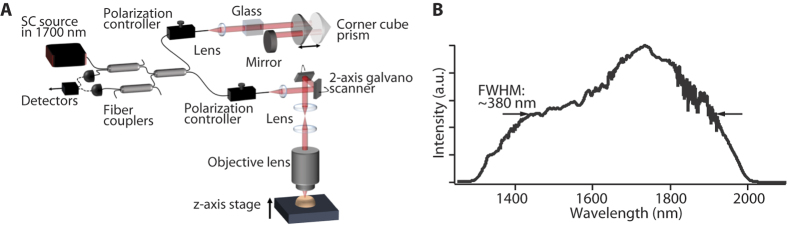
(**A**) Optical setup of the optical coherence microscopy using the SC source in the 1700-nm spectral band and (**B**) optical spectrum of the SC source.

Figure 1A shows a schematic of optical setup for the developed OCM. In this research, to demonstrate the feasibility of high-resolution deep-tissue OCM imaging in the 1700-nm spectral band, we built a time-domain (TD) OCM system with a low-coherence Michelson interferometer using three broadband fiber couplers. Although 1700-nm spectral-domain (SD) OCT systems have already been reported^37^,^38^, it still remains a challenge to achieve high spatial resolution similar to that obtained with the 1700-nm TD-OCT due to the difficulty of broadband aberration correction in a spectrometer. As shown in Fig. 1A, the sample arm of the OCM system consisted of a lens for collimating the output beam from a single mode fiber, two-axis galvanometer scanner (Thorlabs, Inc., GVSM002/M), scan lens, tube lens, and objective lens with a numerical aperture (NA) of 0.65 (Olympus, LCPLN50XIR) and transmittance of 50–60% at around 1700 nm. In the reference arm, a corner cube prism mounted on a galvanometer scanner (Cambridge Technology, Inc., 6240 H) was implemented for optical path length scanning. In this 1700-nm OCM setup, optical glasses were placed in the reference arm to compensate the chromatic dispersion difference between the two arms. For the chromatic dispersion compensation, we first measured the characteristics of the group velocity dispersion of the optics in the sample arm in advance and then chose the combination of glass materials and their thickness. To adjust the polarization states between the two arms, we used the polarization controllers implemented in both arms. The signals from samples were detected with two InGaAs detectors, which have extended wideband characteristics in the 1200–2600 nm wavelength region (Thorlabs, Inc., PDA10D).

Figure 1B shows the optical spectrum of the SC source used for the 1700-nm OCM. The SC source had a monomodal, Gaussian-like spectral shape, which was nearly ideal to achieve a clean interference signal. The center wavelength of the SC source and the spectral width were 1730 and 380 nm, respectively. The output power was 30 mW.

**Figure 2:**
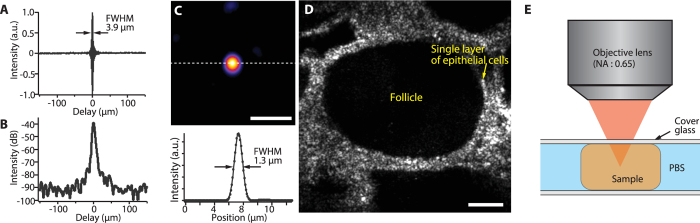
(**A**) Interference signal obtained with the developed OCM, (**B**) the logarithmically demodulated signal, (**C**) OCM image of a single polystyrene bead with a diameter of 200 nm in *x-y* plane (*en*-*face*) and the intensity profile spanning the white dotted line in the image, and (**D**) OCM image of a pig thyroid gland at a depth of 150 μm. (**E**) Sample observation configuration in this experiment. Scale bar: (**C**) 5 μm and (**D**) 50 μm.

To verify the axial resolution of the developed OCM, we measured an interference signal from a mirror (Fig. 2A) and obtained the logarithmically demodulated signal (Fig. 2B). To measure the signal while avoiding saturation of the detectors, the signal power was attenuated by a neutral density (ND) filter, which reduces the signal power by 39 dB in total. From the detected interference signal, we confirmed that the axial resolution of the 1700-nm OCM was 3.9 μm in air, which corresponds to 2.8 μm in tissue under the assumption of a refractive index of n = 1.38. This value was close to the theoretical axial resolution estimated from the spectral width of the SC source (3.48 μm in air and 2.52 μm in tissue). From Fig. 2B, we also confirmed that the sensitivity of the 1700-nm OCM was 93 dB.

The lateral resolution of the developed OCM was theoretically calculated to be 1.21 μm. To evaluate the lateral resolution experimentally, we observed a single 200 nm polystyrene bead embedded in gelatin, which was small enough than the theoretical lateral resolution. As a result, we achieved the OCM image of a single polystyrene bead, and the full-width half-maximum (FWHM) of the intensity line profile was 1.3 μm. This result indicates that the resolving power of the 1700-nm OCM in the lateral direction was close to the theoretical limit. From these experiments, we confirmed that the 1700-nm OCM provides the highest 3D spatial resolution among 1700-nm OCT imaging techniques^32^,^34^,^35^,^36^,^37^,^38^.

To demonstrate *en*-*face* (x-y) OCM imaging of biological tissues, we observed a normal pig thyroid gland. Figure 2D shows the *en*-*face* OCM image of the structures of the pig thyroid gland 0.15 mm below the sample surface. The detail of the image acquisition is described in the method section. As shown in the image, the 1700-nm OCM clearly visualized follicles and a single layer of epithelial cells, which are the characteristic structures of a pig thyroid gland^39^. The incident laser power was 2 mW on the sample. This laser power is below the ANSI damage threshold for skin tissue (9.6 mW for the 1500–1800 nm wavelength region). In this experiment, the sample was fixed by paraformaldehyde to prevent the change of its structures during imaging. To prevent the sample from drying during the observations, we immersed the sample in phosphate buffer saline (PBS) and put a cover glass onto the sample as shown in Fig. 2E.

**Figure 3:**
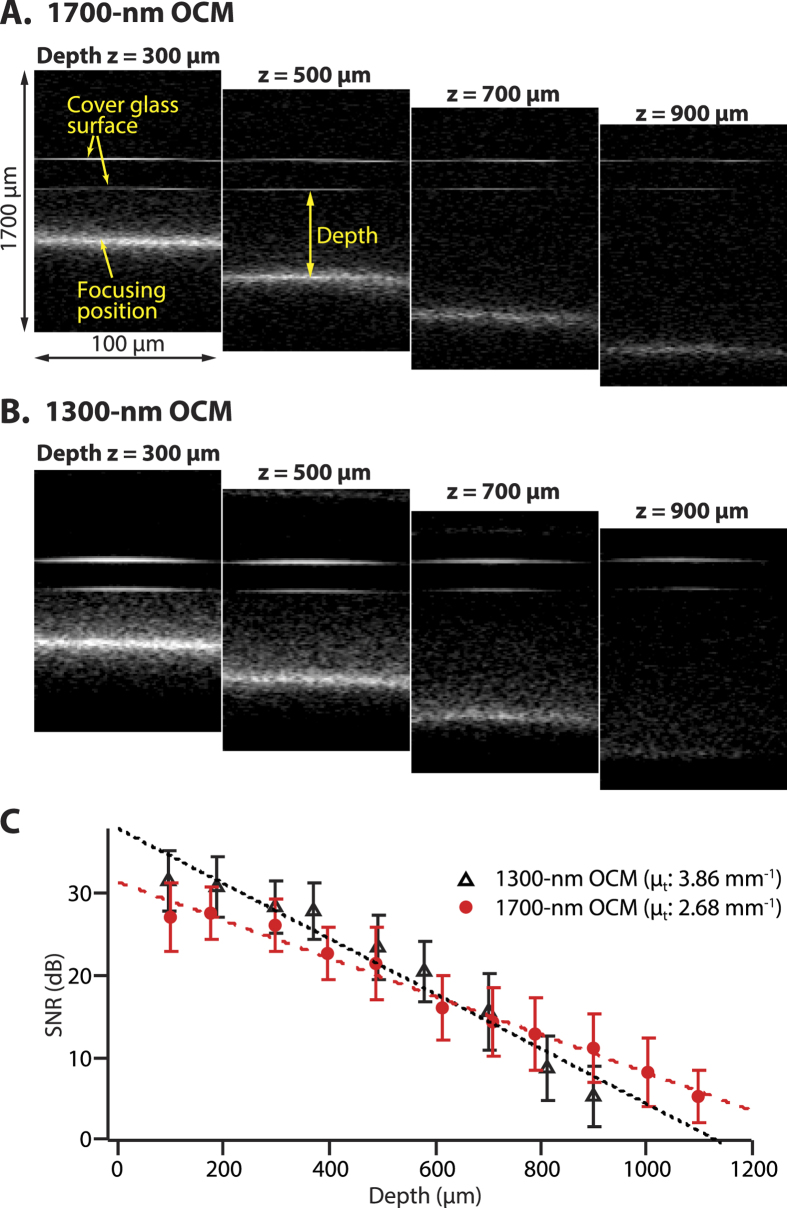
Cross-sectional images of a mouse brain obtained with (**A**) the 1700-nm OCM and (**B**) the 1300-nm OCM. The lateral and axial width of the images were 100 and 1700 μm, respectively. (**C**) SNR of each OCM at difference depths. The dotted lines are exponential fits.

To clarify the enhancement of the OCM imaging depth, we measured the attenuation of signal-to-noise ratio (SNR) with the increase of the imaging depth by using OCM systems in the 1700-nm and 1300-nm spectral bands. To perform the 1300-nm OCM, we switched the 1700-nm SC source to the 1300-nm polarized superluminescent diode (SLD), which has the spectral bandwidth of 45 nm (Thorlabs, Inc., S5FC1018P). The sensitivity for both OCMs were set to be the same by adjusting the light intensity returned from the reference arm by a ND filter. The incident laser power on the sample was 2 mW for both cases. Figure 3A shows cross-sectional images of a mouse brain at the imaging depths of 300, 500, 700, and 900 μm, which were obtained by the 1700-nm OCM. In this experiment, the observation configuration was also the same as that shown in Fig. 2E. Because the back reflection intensity from a cover glass was significantly strong, it was clearly observable even though the focus position of the objective lens was set much further from the position of the cover glass. We defined the imaging depth as the distance between the focus position and the lower surface of the cover glass because the cover glass was placed onto the sample as illustrated in Fig. 2E. Here, to obtain actual physical depths from the optical depth lengths, the refractive index of n = 1.36 was used^40^. After the observations with the 1700-nm OCM, the same cross-sectional plane of the mouse brain was observed by the 1300-nm OCM as shown in Fig. 3B. In our setup, when switching the wavelength from 1700 to 1300 nm, the target sensitivity and axial resolution (14 μm) close to the theoretical one were achieved in the 1300-nm OCM without any additional alignment of the optics in the sample arm. From the results of the cross-sectional images at different focus positions, we obtained SNR at each depth and plotted the SNR values as the function of the depth as shown in Fig. 3C. The OCM system noise levels were determined from the interference signals obtained by measuring reflection light from a mirror. Then, by using a curve fitting based on the exponential decay model (*A*exp(−2*μ*_*t*_*z*)), the attenuation coefficients for the 1700-nm and 1300-nm OCM were evaluated^32^,^33^. Here, *A* is the coefficient, *μ*_*t*_ ( = *μ*_*a*_ + (*1* − *g*) *μ*_*s*_) is the attenuation coefficient, *z* is the depth position, *μ*_*a*_ is the absorption coefficient, *μ*_*s*_ is the scattering coefficient, and *g* is the parameter for the anisotropy of scatterer when multiple scattering is considered^41^. The attenuation coefficients *μ*_*t*_ for the 1700-nm and 1300-nm OCM were 2.68 and 3.86 mm^−1^, respectively. In this measurement, we confirmed that *μ*_*t*_ for the 1700-nm was 1.44 times smaller than that for 1300-nm. This result clearly indicates that the 1700-nm OCM offers larger imaging depth than the 1300-nm OCM under the condition of the same sensitivity. This difference of *μ*_*t*_ values is similar to that reported in the literature^41^.

**Figure 4:**
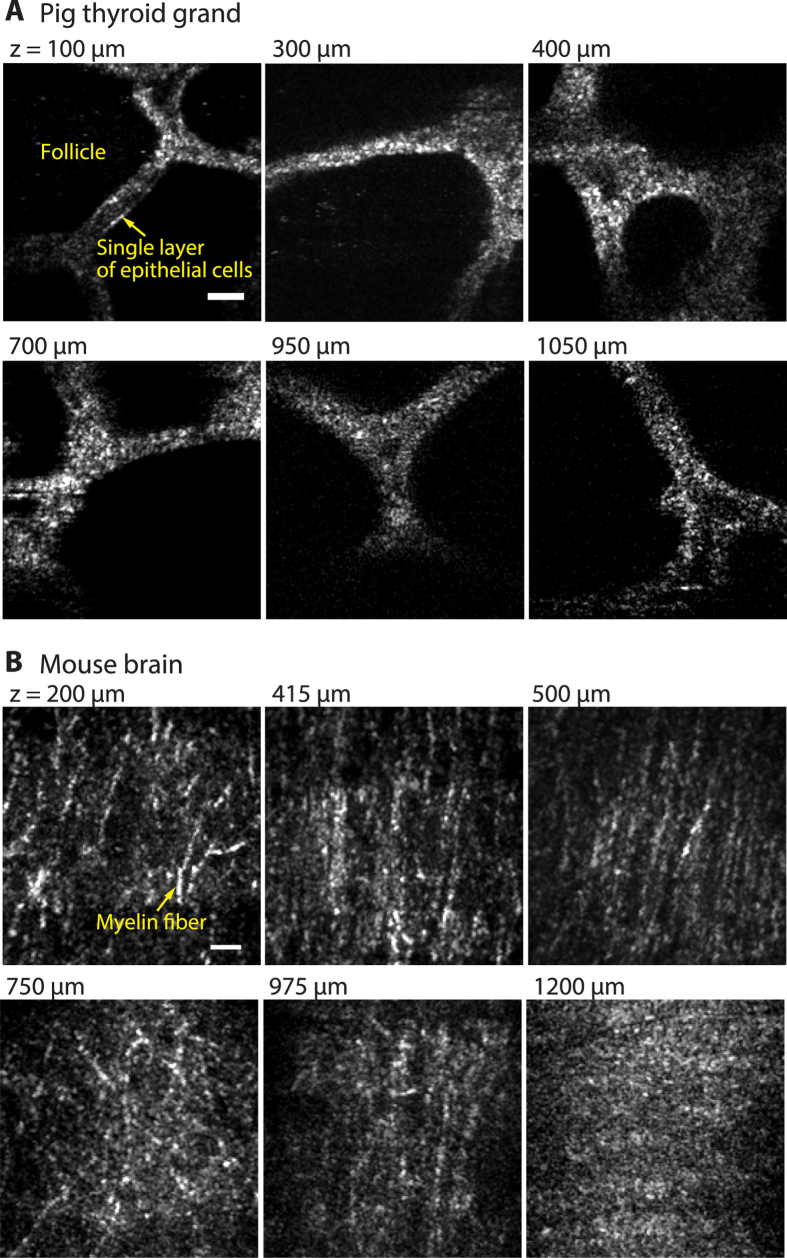
OCM images (*x*-*y* plane (*en*-*face*)) of (**A**) a pig thyroid grand and (**B**) a mouse brain at different imaging depths. Scale bar: 20 μm.

We then performed *en*-*face* OCM imaging of a fixed normal pig thyroid gland and raw mouse brain at various imaging depth. In imaging of a normal pig thyroid gland, follicular structures were visualized with enough SNR up to a depth of 1.05 mm (Fig. 4A). Figure 4B shows the *en-face* OCM images of a mouse brain. In the observations of the mouse brain, we performed 10-μm maximum intensity projection along the axial direction (*z*-direction) to enhance the visibility of structures in the mouse brain. As shown in the figure, fiber like structures were clearly observed up to a depth of 0.975 mm (Fig. 4B). The fiber like structures are considered to be myelin fibers, because myelin fibers (myelinated axons) are strong light scatterers due to the high refractive index of the lipid-rich myelin sheath and it has already been reported that OCM is able to observe myelin fibers^20^. At the depth of 1.2 mm, almost no fiber like structures were seen in the OCM image. According to the results reported previously in refs 8, 20 and 42, it is considered that this image displays structures of white matter or alveus hippocampi in the mouse brain. From these results, we confirmed that the 1700-nm OCM allows us to obtain OCM images with high SNR up to a depth of 1.2 mm. In this experiment, the imaging depth was confirmed by the same way as the experiments in Fig. 3. To achieve the actual physical depth, we used the refractive index of the samples (n = 1.36 for a mouse brain^40^ and 1.38 for a pig thyroid gland).

## Discussion

In this study, we demonstrated the deep-tissue imaging capability of the 1700-nm OCM. Although the comparison of the attenuation coefficient in Fig. 3 clearly indicates that the 1700-nm OCM has the lower attenuation coefficient than the 1300-nm OCM, the current imaging depth in the 1700-nm OCM was similar to that obtained in the previous report of 1300-nm OCM^22^. The main limiting factor is the relatively low sensitivity in the 1700-nm OCM. As shown in Fig. 2B, the sensitivity of the 1700-nm OCM was 93 dB. On the other hand, the sensitivity of 105 dB was achieved for the 1300-nm OCM in the literature^20^. This relatively low sensitivity in the 1700-nm OCM is mainly due to three factors. The first factor is the high noise property of the extended InGaAs detector used for the detection of the 1700-nm spectral band^37^. The second one is the wide electronic detection bandwidth to take full advantage of the spectral bandwidth (FWHM: ~380 nm) of the SC source. Because the white noise components distribute in the entire temporal frequency region, the wider the electronic detection bandwidth in a time-domain scheme becomes, the higher the noise level becomes^43^. Here, we confirmed that, when the electronic detection bandwidth was reduced to around 30–45% in our setup, the noise level dropped by 4–6 dB. The third one is the use of a time-domain scheme for the 1700-nm OCM. For the 1300-nm OCM in the literature^20^, a SD-OCT scheme was utilized. It has been reported that a Fourier domain (FD) OCT, including SD-OCT and SS-OCT, offers higher sensitivity than a TD-OCT^43^. In addition, the issue of the electronic detection bandwidth would also be bypassed by using the FD-OCT scheme^43^. Therefore, if a FD scheme is implemented into the 1700-nm OCM, higher sensitivity would be achieved.

In addition to the sensitivity issue in the 1700-nm OCM, the use of non-immersion objective lens is also one of the current limiting factors of the imaging depth. It is well known that aberration effects in focusing light into samples appear severely when the difference of the refractive index of samples and mediums between the focusing lens and samples are large^44^,^45^,^46^. The aberrations, such as spherical and chromatic aberrations, cause the spread and blurring of the light focus and reduces the light intensity at the light focusing position, resulting in the decrease of SNR in imaging. In our experiment, we used the non-immersion objective lens with correction collar, and adjusted the correction collar so that the OCM signal intensity was maximized when the observed depth position was changed. However, because the magnitude of the aberration is quite large due to the large refractive index mismatching between air (n = 1) and tissues (n = ~1.38) and it becomes larger with the increase of the imaging depth, it was not possible to reduce the aberration effects by using only the correction collar at a depth over the current maximum imaging depth shown in Fig. 4. In current deep tissue imaging, a water-immersion objective lens is the first choice in most cases (the refractive index of water: n = 1.33). The small refractive index mismatching between water and tissues reduces the magnitude of the aberration. Therefore, compared to the case in using non-immersion objective lenses, it is possible to achieve higher SNR by using a water-immersion objective lens for tissue imaging, resulting in the enhancement of the imaging depth^44^,^45^,^46^. The use of such objective lenses for the 1700-nm OCM would realize further improvement of the imaging depth. Unfortunately, however, such immersion objective lenses with a high NA and high transmittance from 1400 to 2000 nm wavelength region have not been commercially available at this moment.

In addition to the efforts of implementation of the longer wavelength, the way based on adaptive optics have also been intensively studied to improve the imaging depth^47^,^48^,^49^. By using the approaches, it is possible to achieve less spread and blurred light focus at the observation position in tissues by correcting the incident light wavefront distorted by multiple light scattering, spherical aberration, chromatic aberration, and so on. So far, adaptive optics techniques with some optical devices such as digital micro-mirror devices have been proposed and demonstrated biological imaging with the improved imaging depth^47^,^48^,^49^. The implementation of these approaches to the 1700-nm OCM would also help the further enhancement of the imaging depth.

In our observation of a pig thyroid gland, the sample was formalin-fixed to avoid artifacts by the change of the structures during the observations. Although the formalin fixation is quite powerful to keep sample morphology, it has been reported that formalin fixation process increases the scattering coefficient by protein cross linkage, sample hydration, and shrinkage^50^. The absorption coefficient would also increase if chromophores are not damaged during the fixation process. In the literature, ~10% increase of the attenuation coefficient was reported in a rabbit mouse^50^. The imaging depth would be slightly reduced if the samples are formalin-fixed.

Recently, the number of reports about imaging techniques in the 1700-nm spectral band has been increasing^32^,^34^,^35^,^36^,^37^,^38^,^42^. This fact points out that this spectral window has already been recognized as a promising choice for biomedical imaging. Considering the circumstance, a variety of imaging devices for this wavelength region would be commercially available in the near future, and this wavelength region would become one of main choices for biomedical imaging.

In summary, we demonstrated optical coherence microscopy using the 1700-nm spectral band for deep-tissue imaging with high spatial resolution. Thanks to the use of the ultrabroad SC source in the 1700-nm spectral band, both the high 3D spatial resolution and the large imaging depth up to 1.2 mm was realized in imaging animal tissue samples. Our results indicate that the 1700-nm OCM has the potential to enhance the imaging depth of high-resolution OCM.

## Methods

### Generation of the ultrabroad SC in the 1700-nm spectral band

For the 1700-nm OCM, we employed our previously developed SC source in the 1700-nm spectral band^34^,^35^,^36^. In this SC source, mode-locked ultrashort pulse Erbium (Er) doped fiber laser at a center wavelength of 1560 nm (IMRA femtolite B5) was used as the seed pulse and then the seed pulse was amplified by an Er-doped fiber amplifier. The chirp of the amplified seed pulse was compensated by a large-mode-area photonic crystal fiber. To generate the SC source shown in Fig. 1B, the chirp compensated pulse was coupled into a highly nonlinear fiber with a normal dispersion property. This approach provides an SC source with low-noise properties because the SC source has been generated through self-phase modulation only.

### Polystyrene bead sample preparation

Gelatin powder was solved in distilled water (gelatin: 10 wt%). Then, polystyrene bead with a diameter of 200 nm (Polyscience, Inc., 07304-15) was mixed with the gelatin-water solution and the final mixture was solidified at room temperature.

### Pig thyroid gland and mouse brain sample preparation

In our experiments, we used fixed pig thyroid glands and raw mouse brains as samples. We purchased both adult pig thyroid gland and mouse brain from a company. Both were delivered in cold storage (4 degree Celsius) immediately after surgical removal of the organs from pig and mouse. Then, the pig thyroid gland was fixed by paraformaldehyde because the inner follicular structures easily changed under unfixed condition. During the measurements, samples were immersed in phosphate-buffer saline solution to prevent morphological changes by drying.

### Image acquisition and construction of *en-face* (x-y) OCM images

The scanning method was the same as that of a standard TD-OCT with a two-axis galvanometer scanner for x-y axis (2D lateral direction) scanning, which is a depth priority technique^51^. In our OCM imaging, firstly, the focus position was set by a single-axis motorized stage (Sigma Tech, Inc., FS-1050SP), which was mounted vertically against an optical table to move the focus position along the z (axial)-direction. A-scan was achieved by scanning the optical path length with the galvanometer mirror (Cambridge Technology, Inc., 6240 H) in the reference arm. Then, B-scan was achieved by taking multiple A-scans through x-axis scanning by the galvanometer scanner (Thorlabs, Inc., GVSM002/M) in the sample arm. Finally, a 3D volume dataset was constructed by multiple B-scans through y-axis scanning by the galvanometer scanner in the sample arm. After image acquisition, the signal intensity data in the focus position was extracted from the 3D volume dataset to obtain an *en-face* (x-y) OCM image. To generate *en*-*face* OCM images without those images blurred, it was necessary to compensate the scan delay curvature^51^, because the optical path length became different in each scanning angle. The position data of the scan delay curvature was extracted by tracking the peak signal of the 3D volume dataset obtained from coverslip measurement. Then, the position data was referred to extract a compensated *en-face* OCM image from the 3D volume dataset of the interest object. In this experiment, we used the refractive index of the samples to calibrate the path length. In constructing OCM images, a bilateral filter was applied to suppress speckle noise^52^,^53^. We also applied a square root compression to OCM images for visualization^54^. Therefore, all *en-face* OCM images in this paper are displayed in a square root scale.

## Additional Information

**How to cite this article**: Yamanaka, M. *et al*. Optical coherence microscopy in 1700 nm spectral band for high-resolution label-free deep-tissue imaging. *Sci. Rep*. **6**, 31715; doi: 10.1038/srep31715 (2016).
